# Health-Related Productivity Loss According to Health Conditions among Workers in South Korea

**DOI:** 10.3390/ijerph18147589

**Published:** 2021-07-16

**Authors:** Dong-Wook Lee, Jongin Lee, Hyoung-Ryoul Kim, Mo-Yeol Kang

**Affiliations:** 1 Department of Preventive Medicine, College of Medicine, Seoul National University, Seoul 08826, Korea; taesanglee89@gmail.com; 2 Department of Occupational and Environmental Medicine, Seoul St. Mary’s Hospital, College of Medicine, The Catholic University of Korea, Seoul 07345, Korea; leejongin.md@gmail.com (J.L.); cyclor@catholic.ac.kr (H.-R.K.)

**Keywords:** productivity loss, health status, health conditions, absenteeism, presenteeism

## Abstract

This study aimed to investigate the degree of health-related productivity loss (HRPL) for common health conditions. A total of 4197 workers participated in a web-based questionnaire survey from January to February 2020. HRPL was measured using the Work Productivity and Activity Impairment questionnaire, and a difference in HRPL was calculated for each common health condition. The burden of productivity loss due to each health condition was calculated by the product of the difference in HRPL scores and the percentage of participants who complained. The health conditions most strongly associated with increased HRPL were infertility treatment (30.6%), osteoporosis (25.9%), cancer (25.3%), gastric ulcer or duodenal ulcer (25.0%) and anaemia (23.9%). The most important health conditions in order of their magnitude of induced burden of productivity loss were fatigue, neck or shoulder pain, insufficient sleep, back pain, headache, common cold and flu, insomnia, anxiety and diarrhoea or constipation. HRPL is more strongly and importantly associated with the aforementioned health conditions. Occupational health managers should prioritise addressing health conditions strongly and importantly associated with HRPL when implementing health promotion programmes.

## 1. Introduction

Workers’ health status is a fundamental factor influencing the improvement and maintenance of productivity by the labour force [1,2]. Since the late 1970s, the relationship between health conditions and workplace outcomes has been the basis of the workplace health management field, and employee health has become a major focus of businesses worldwide, as employers have begun to recognise employees as being an essential asset, and investment in their health as being essential to maintain competitiveness [3]. Currently, there is consensus that the creativity and productivity of the workforce is the driving force for corporate success. In this regard, health can be assumed as an element of human capital within a society where investments to improve worker’s health are reflected in the economic growth [4].

Many studies have revealed a positive relationship between workers’ health status and health-related costs. In the workplace, the economic burden of poor health includes not only medical and pharmaceutical expenses but also health-related productivity loss (HRPL) due to sick leave (absenteeism) and reduced performance while at work as a result of uncontrolled diseases or health risks (presenteeism). Several research studies have shown that the financial cost of absenteeism and presenteeism outweighs medical and pharmaceutical expenses [5,6,7].

In the field of health and productivity, several studies have investigated the impact of various diseases and health risks on HRPL [6,8,9,10]. Employees with high absenteeism and presenteeism reported poorer physical health conditions. These include heart disease, migraines, asthma, kidney disease and diabetes. In terms of mental health, job stress, depression and burnout are associated with higher HRPL. On the other hand, lifestyle factors such as sleep disorders and smoking are similarly associated with increased HRPL. Conversely, the higher the self-reported job satisfaction and organizational commitment to employees’ well-being, the lower the HRPL.

From a societal perspective, for the best allocation of limited societal resources, it would help to understand the comparative burden of HRPL according to specific health conditions more comprehensively. Evidence on impaired productivity caused by certain health problems among workers can also motivate employers to change work conditions and practices (e.g., employee benefits, support services and facilities) for employees with different health problems to further reduce productivity loss and retain skilled workers longer in the workforce. However, while there have been studies focused on measuring the number of absent workdays among workers with one health condition [11,12,13,14,15], only a few have examined the comparative burden of different commonly occurring health conditions on labour productivity [16]. Therefore, this study aimed to evaluate and compare the HRPL according to workers’ common health conditions.

## 2. Materials and Methods

### 2.1. Study Participants

Study participants were recruited between 6 January and 18 February 2020, and data were acquired via a web-based questionnaire through Panelnow (https://www.panelnow.co.kr, accessed on 22 May 2021), an online panel survey service operated by DataSpring Korea Inc. (Seoul, South Korea). We targeted employees aged 19 years or older in South Korea, and a total of 4197 participants were randomly recruited. After excluding non-waged workers (*n* = 315), our final analysed sample was composed of 3882 waged workers in South Korea. As the survey was performed through an online system that ensured completeness of the questionnaires, there was no missing information in the completed data of the study participants. This study was approved by the institutional review board of the Catholic University of Korea Seoul St Mary’s Hospital (KIRB-20200219-014).

### 2.2. Health Conditions

The participants’ various health conditions were assessed using a questionnaire item as follows: ‘Here is a list of frequent health conditions among workers. Please check if you are currently affected (multiple choice)’. The list of health conditions was selected based on a previous study related to our objectives [6]. The health conditions included the following: respiratory symptoms, liver disease, diabetes mellitus, dyslipidaemia, hypertension, cardiac disease, cancer, anaemia, osteoporosis, allergic disease, skin disease or itching, asthma, the common cold or flu, enterocolitis, gastric ulcer or duodenal ulcer, diarrhoea or constipation, esophagitis, upper limb or lower limb pain, back pain, neck or shoulder pain, headache, dental problems, depression, anxiety, insomnia, sleep deprivation, post-menopausal or post-andropausal symptoms, infertility treatment, fatigue, having a hangover, hearing impairment, eye disorders, urinary symptoms and any health condition not listed.

### 2.3. Measurement of Health-Related Productivity Loss

For the purpose of measuring the loss of labour productivity, several measurement tools are typically used to assess absenteeism due to health in paid working days. However, individuals could have an inadequate status to work due to their healthy status in working days, whether paid or not [17]. Considering these limitations, we assessed HRPL using the Work Productivity and Activity Impairment questionnaire, general health version (WPAI:GH) [18]. The WPAI:GH comprises six items assessing: current employment status (Q1), number of work hours missed due to ill health in the past seven days (Q2), hours of work missed for other reasons such as vacation(Q3), actual number of hours worked in the past seven days (Q4), extent to which a health problem affected productivity at work (Q5, ranging from 0 to 10) and extent to which the health problem affected daily activities excluding work (Q6, ranging from 0 to 10). Productivity loss due to absenteeism, which is measured as the percentage of work time missed due to health problems in the past seven days, is calculated as follows: ‘hours missed from work because of health problems in the last week (Q2)/(hours missed due to health problems [Q2] + hours worked in the last week [Q4])’. Productivity loss due to presenteeism, which is measured as the percentage of impairment experienced at work due to health problems in the past seven days worked in the last week (Q5/10), is calculated as the percentage of productivity loss due to health problems in the last week. The overall HRPL percentage is calculated as ‘productivity loss due to absenteeism (Q2/(Q2 + Q4)) + productivity loss due to presenteeism (Q5/10) × (1 − productivity loss due to absenteeism) (1 − (Q2/(Q2 + Q4))’. The validity and reliability of the WPAI:GH has been verified [18], and a Korean version of the questionnaire was developed through independent translations, harmonisation, back-translation, an expert review and a review by local language users carried out by the tool developer [19].

### 2.4. Measurement of Other Variables

Household income was assessed using the following question: ‘What was [your] approximate gross household income over the last year, including labour income, real estate income, pensions, interest income, public income transfer and private transfers from relatives and your family?’. We classified participants into three groups according to household income terciles. Meanwhile, information on the participants’ age, gender, educational level (i.e., high school and below, college or university or graduate school), marital status (i.e., single, married, separated, widowed or divorced), employment status (i.e., regular, temporary or day labourer), job classification (i.e., white collar or blue collar), weekly working hours (i.e., <40, 40, 41–52 or >52 h/week), shift work status (i.e., no vs. yes), smoking status (i.e., no or yes), binge drinking (i.e., ≥7 standard drinks for men or ≥5 standard drinks for women at least once a month or not) [20] and exercise (i.e., moderate exercise more than 150 min/week or not) were collected through the questionnaires.

### 2.5. Statistical Analysis

It was calculated that the ratio of HRPL due to a specific health complaint among 34 listed health conditions to HRPL of workers who did not complain about any health conditions (*n* = 968, 24.9%). As individuals can report more than one health condition, workers who had complained of various symptoms or diseases were included in the multiple-health-conditions group and compared with the group of workers without any health conditions. For example, data of person who complained about fatigue and hypertension were used both when comparing a group of workers with fatigue to workers without any health conditions and when comparing a group of workers with hypertension to workers without any health conditions. As 29.1% of participants showed HRPL as zero percent, and outcome variables are skewed with many zeros (Supplementary Figure S1), we adopted negative binomial regression models to calculate the ratio of HRPL of participants with a specific health condition to that of participants without any health conditions [21]. Negative binomial regression models were constructed for each health condition, and a coefficient for the health condition (coded as 0 [without the health condition] or 1 [with the health condition]) was calculated as the ratio of HRPL according to the health condition. Negative binomial regression models were constructed adjusting for age, gender, educational level, household income, marital status, employment status and weekly working hours. As we constructed 34 models for each health condition, Bonferroni correction was used to consider the multiple testing issue. Additionally, we calculated the ratios after stratifying for gender. Next, we calculated the ratio of absenteeism and presenteeism of workers with the specific health condition to those of participants with no complaints. Finally, we estimated the burden of HRPL for each health condition as the product of the percentage of participants who had a specific health condition and the HRPL ratio of the health condition, which were calculated through a negative binomial regression analysis. All statistical analyses were performed using the software SAS (version 9.4; SAS Institute, Cary, NC, USA) and R version 3.4.4 (R Foundation for Statistical Computing, Vienna, Austria). Two-tailed *p*-values < 0.0014 were considered to indicate statistical significance, as Bonferroni correction was adopted to consider multiple testing for the 34 models.

## 3. Results

Table 1 shows the demographic characteristics of the study participants. Of the 3882 waged workers, 1944 (50.1%) were males, and 1938 (49.9%) females. The average percentage of HRPL was 26.6% (SD ± 26.7), the percentage of participants with HRPL of 0 was 29.1%, and the percentages of HRPL for males and females were 24.5% (SD ± 25.6) and 28.7% (SD ± 27.5), respectively. The percentage of HRPL was higher for the young and the low-income workers and for those who had separated. The demographic characteristics of the study participants by health conditions are presented in Supplementary Table S1.

**Figure 1:**
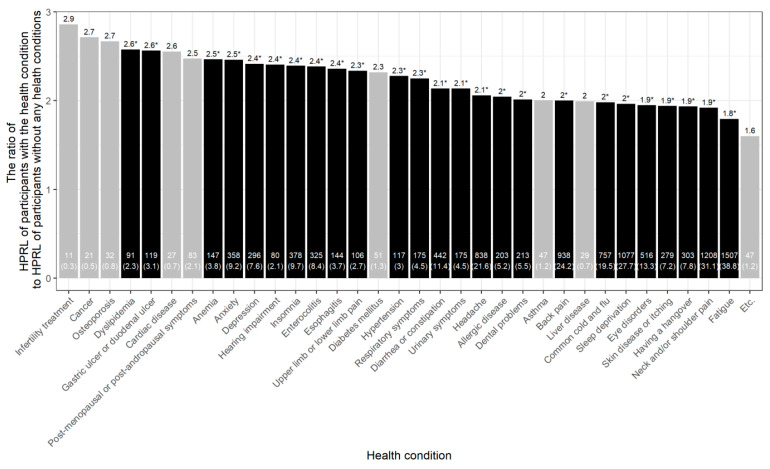
Percent health-related productivity loss among workers with specific health conditions compared to that of participants without any health conditions. The number and percentage of workers who complained of specific health conditions are presented at the bottom of the bar plot. The ratio of HRPL loss between participants with specific health conditions and that between healthy participants (without any health conditions) was calculated with the adjustment for age, gender, education level, household income, marital status, employment status and weekly working hours. * *p* < 0.0014 was in black.

Figure 1 shows the prevalence of each health condition among participants, and the ratio of HRPL of participants with a specific health condition to HRPL of the participants without any health conditions. The most common health conditions among the participants were a sense of weariness or fatigue (*n* = 1507, 38.8%), neck and/or shoulder pain (*n* = 1208, 31.1%), insufficient sleep (*n* = 1077, 27.7%), back pain (*n* = 938, 24.2%) and headaches (*n* = 838, 21.6%). The top five health conditions that significantly affected HRPL were dyslipidaemia (×2.58, *p* = 0.0002), hypertension (×2.56, *p* = 0.0002), anaemia (×2.47, *p* < 0.0001), anxiety (×2.46, *p* < 0.0001) and insomnia (×2.39, *p* < 0.0001). Supplementary Figure S1 and Supplementary Table S2 summarise the results stratified by gender. 

Supplementary Table S3 presents two components of HRPL, i.e., absenteeism and presenteeism, according to health conditions. The health conditions that significantly affected absenteeism were headache (×2.77, *p* = 0.0002), common cold and flu (×3.16, *p* < 0.0001), insomnia (×3.69, *p* = 0.0005) and anxiety (×4.03, *p* = 0.0004). The top five health conditions that significantly affected presenteeism were gastric ulcer and duodenal ulcer (×2.58, *p* < 0.0001), dyslipidaemia (×2.53, *p* = 0.0004), anxiety (×2.47, *p* < 0.0001), anaemia (×2.44, *p* < 0.0001) and hearing impairment (×2.40, *p* = 0.0009).

**Figure 2:**
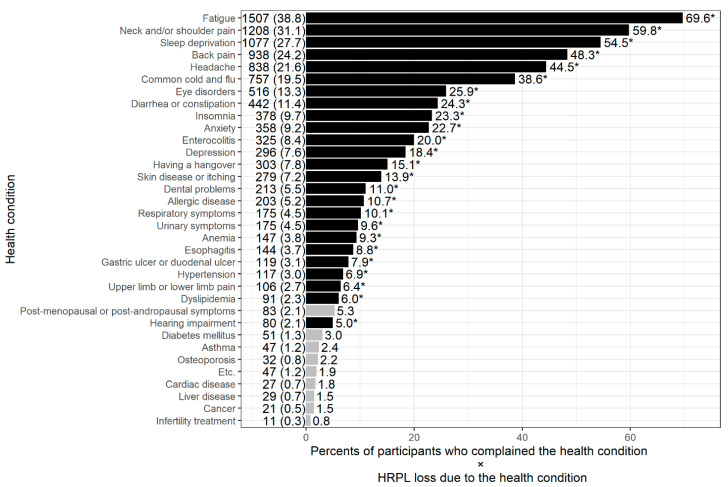
Work impairment burden of health conditions compared to that of participants without any health conditions. The number and percentage of workers who complained of specific health conditions are presented on the left side of each bar plot. The ratio of HRPL loss between participants with specific health conditions and that between healthy participants (without any health conditions) was calculated with adjustment for age, gender, education level, household income, marital status, employment status and weekly working hours. * *p* < 0.0014 was in black.4.

Figure 2 illustrates the burden of HPRL induced by specific health conditions, the product of the HRPL percentage due to each health problem and the proportion of participants with a specific health problem. The health conditions that affected work impairment burden the most were fatigue (69.6), neck and/or shoulder pain (59.8), sleep deprivation (54.5), back pain (48.3), headache (44.5), the common cold and flu (38.6), eye disorders (25.93), insomnia (23.3), anxiety (22.7) and enterocolitis (20.0). Health conditions for the heavy burden of work impairment due to health did not obviously differ according to gender (Supplementary Figures S3 and S4).

## 4. Discussion

This study primarily sought to examine and compare the association between different health conditions and labour productivity loss among the Korean working population. We found every health condition we investigated to be significantly associated with HRPL. Overall, the top six health conditions were fatigue, neck or shoulder pain, insufficient sleep, back pain, headache and common cold or flu.

In the field of occupational health and policy, we believe that it is necessary to explore which health condition has the biggest impact on productivity to set priorities for problems and plan an efficient allocation of resources. However, there have been only a few studies on the association between specific health conditions and HRPL. A recent study involving 3258 workers in the USA reported that presenteeism contributed to 93.6% of the annual productivity loss by using WPAI, but the study did not report which health condition is important for productivity loss [10]. A study involving 5583 USA workers reported that musculoskeletal problems (back pain and foot and leg problems) were significantly associated with productivity loss, but assessed productivity loss only by using a simple question (whether people’s health limited their work or not) and did not ask about the severity of the disease [22]. A study with data from the Netherlands Working Conditions Survey reported that musculoskeletal complaints and psychological complaints were associated with low performance at work; this study also assessed health conditions by asking employees whether they experienced one or more conditions in a not severe manner [23]. In line with previous studies, our study reported that musculoskeletal disease (painful neck or stiff shoulders, back pain) and psychological complaints (insomnia and anxiety) are the most common and important health issue for workers’ productivity. Furthermore, fatigue and insufficient sleep were suggested as the occupational health problems that must be dealt with first. Our study emphasizes the need of prioritizing health issues of workers related to productivity loss in order to plan occupational health programs for them. Our findings match those from earlier studies. In a Canadian study examining the association between different chronic health conditions and absence in workdays using representative, population-based survey data, 10 out of 16 chronic diseases (asthma, arthritis, back pain, diabetes, COPD, migraines, heart disease, cancer, mood disorders and bowel disorders) were reported to be significantly associated with increased absenteeism due to health problems [16]. Overall, mood disorders, heart disease, intestinal disorders, back pain and cancer caused the highest increases in health-related absenteeism at the worker level, while, at the national level, the largest productivity losses were associated with back pain, mood disorders, migraines, bowel disorders and arthritis. In a study of various staff under the US healthcare system, the highest daily loss of productivity and annual cost per capita were linked to migraine or severe headache, chronic back and neck pain and mental illness such as generalised anxiety and depression [24]. More recently, a Japanese study found that experiencing a stiff neck or shoulders, fatigue, depression or anxiety and dry eye problems accounted for a large proportion of sicknesses among employees [8]. Loeppke et al. studied the medical and pharmacy claims reported by 10 employers regarding 51,648 employees in the USA and showed that health-related productivity loss was significantly associated with medical and pharmacy costs [5]. A cross-sectional study in Japan similarly investigated absenteeism and presenteeism and related medical and pharmaceutical expenses by questionnaires and reported that total cost burdens associated with chronic illness were associated with musculoskeletal and mental health disorders [6]. We found similarly that the magnitude of HRPL burden was significant due to fatigue, musculoskeletal pain, sleep, headache, eye disorders and mental diseases in our study, though we just considered absent time from workplace due to health and work productivity loss due to presenteeism during participants’ work time.

Fatigue is one of the most common concerns among workers and a well-established predictor of sickness absenteeism and presenteeism. It may play a mediating role between suboptimal health conditions and HRPL [25] because it is a general term used to describe a wide variety of conditions. Fatigue is difficult to quantify because it refers to a subjective and personal experience [26]. Symptoms of fatigue can include muscle weakness, lethargy, inability to think clearly or concentrate, apathy, cognitive decline, anxiety and exhaustion. Lack of recovery from work due to long working hours may lead to work-induced chronic fatigue, which is irreversible and no longer responds to physiological compensation mechanisms [27]. Consequently, the risk of ill health, absenteeism, and presenteeism may increase. If a worker has also lost sleep, fatigue and sleepiness could combine to worsen workers’ health conditions and their labour performance. 

Musculoskeletal pain, such as neck or shoulder pain and back pain, showed an overall large burden of HPRL in Korean workers (Figure 2). Although musculoskeletal pain is very common among workers, studies in the literature focusing on productivity loss and musculoskeletal injuries are surprisingly sparse. Hagberg et al. reported that the average decrease in productivity due to diseases of the musculoskeletal system is about 15% for women and 13% for men [28]. Meerding et al. also reported that the average productivity loss for industrial workers is 7%, and that for construction workers with musculoskeletal diseases is 25% [29]. It has also been shown that workers with osteoarthritis complaints during work time reported an average of 9% productivity loss [30]. Meanwhile, a telephone survey of nearly 29,000 working adults using the Work and Health Interview questionnaire estimated that chronic pain from headaches, arthritis, back pain and other musculoskeletal problems caused 13% of productivity loss among the US workforce at a cost of $62.1 billion per year [31]. A total of 76.6% of these costs were attributed to presenteeism, and the rest to absenteeism. Although these studies have merit, more research in this area needs to be undertaken in various workplace settings to ascertain the impact on employers.

An acute illness, such as the common cold or flu, might reduce an individual’s ability to work, as well as labour productivity. Under acute conditions, employees decide whether to attend work on a day they do not feel well. Many factors may influence such a decision, including the availability of effective medication to treat their symptoms, their ability and motivation to work [32] or the demands placed on them at work that day. Under certain public health crises, such as the ongoing COVID-19 pandemic, however, workers with flu symptoms should not attend work and should instead delay returning to physical workplaces until their lack of infectivity has been proven [33]. In these cases, productivity loss to some degree is inevitable, although one can work at home.

Apart from physical health, mental health has also been shown to affect absenteeism and presenteeism. Workers with psychological complaints were more likely to show poor performance at work, and such employees may not be easy to cope with at work. A systematic review showed that the association between mental health and decreased productivity has been ascertained consistently in studies on sickness presenteeism and mental health [34]. For instance, a longitudinal study that measured the influence of health on output at work found that depression and anxiety had substantial and persistent negative effects on labour productivity [35]. These results are accordant with the findings of the current study showing that insomnia, anxiety and depression were associated with productivity loss by 20.1%, 20.4% and 21.5%, respectively (Figure 1).

This study benefited from the use of a large diverse sample with almost 4000 participants to explore the relative influence of health-related factors on productivity loss among workers in Korea. Additionally, the use of an online survey allowed us to better generalise the results of this study by efficiently capturing data from a larger and more diverse population.

At the same time, however, this study has several methodological weaknesses. First, its cross-sectional design prevents causal inferences from being made about the reported relationship between health conditions and productivity loss. In addition, selection effects, due to the fact that those who had severe diseases quit their jobs before the start of the survey, should be acknowledged. Second, although the data collection was based on the panel participants randomly selected, the sample estimate has a limitation in its lack of being reflective of the actual conditions of all workers in the country. For example, the percentage of wage workers aged 20–29, 30–39, 40–49, 50–59 and 60 years were 13.1%, 23.9%, 26.4%, 22.7% and 13.9%, respectively, in the nationally representative survey in South Korea [36,37], but our data were obtained from a higher proportion of younger workers. However, we believe that the important health conditions suggested by our study are of significant importance for the entire working population in South Korea. Third, absenteeism and presenteeism were measured using self-administered questionnaires that were answered only once; thus, the seasonal effect or episodic nature of some conditions such as allergy to pollen, common cold and flu would not be randomly distributed in the data. The survey was conducted in winter; thus, flu and common cold had a higher prevalence rate, which may have led to an overestimation of their true burden. This should be considered in the interpretation of the results. Finally, health conditions were simply determined on the basis of self-assessed questionnaires, so the reliability of the reported health conditions can be limited. As we asked participants which health condition affects their work performance, the severity or duration of health conditions was not assessed in our study. To overcome these limitations, further analyses using hospital register data and/or national health insurance claim data may be warranted.

This study has implications for employers to maintain their workplace productive. The results regarding productivity loss due to different health conditions can aid employers prioritise employee health management and develop effective workplace health and human capital investment strategies. Implementing screening and educational programmes for workers to prevent undiagnosed or subclinical illnesses should allow workers to better manage their own health conditions. The World Health Organization’s health promotion model in the workplace suggests that the first task of a team for a healthy workplace should be to assess the present situation and desired future conditions for both the enterprise and the workers [38]. In this context, evaluating the prevalence and magnitude of the problem that affects HPRL is the most important task to implement health promotion programmes. 

The findings of this study also indicate a need for evidence-based policies from a society perspective. Allocating limited healthcare resources would improve the quality of life and life expectancy of the citizens in a society. For this, policymakers need information on the economic impact of health problems and the return on investment of health interventions. While it is important that workers’ health promotion programmes target the highest-risk workers and the groups with the highest direct and indirect costs, it is equally important to provide programme opportunities to the majority of workers at medium- or low-risk of health problems. Taking a more comprehensive management approach can prevent healthy employees from becoming high-risk workers in the future. Maintaining workers’ good health is always the most effective health strategy [34].

## 5. Conclusions

Health conditions, especially fatigue and insufficient sleep, musculoskeletal pain, the common cold or flu and mental health problems are associated with substantial labour productivity loss. These findings can aid both health managers in a company and occupational health policymakers in prioritising workers’ health problems and developing measures for the prevention and management of these health conditions, which will allow them to minimize the HRPL from absenteeism and presenteeism. Beyond our findings, future studies should focus on the cost–effectiveness or cost benefits of workers’ health promotion programmes from a total (direct and indirect) cost perspective.

## Figures and Tables

**Table 1:** Demographic characteristics and health-related productivity loss (%) of the study participants.

	Total			Male			Female		
		HRPL (%)			HRPL (%)			HRPL (%)	
	*n* (%)	Mean	SD	*n* (%)	Mean	SD	*n* (%)	Mean	SD
Total	3882 (100)	26.6	26.7	1944 (50.1)	24.5	25.6 **	1938 (49.9)	28.7	27.5
Age									
20–29	948 (24.4)	30.0	27.6 **	400 (20.6)	27.3	26.9 *	548 (28.3)	32.0	27.9 **
30–39	1098 (28.3)	28.5	26.7	565 (29.1)	25.1	25.5	533 (27.5)	32.1	27.5
40–49	1117 (28.8)	25.0	26.5	584 (30)	24.2	25.8	533 (27.5)	26.0	27.2
50–59	522 (13.5)	21.8	24.8	278 (14.3)	21.7	23.4	244 (12.6)	21.8	26.3
≥60~	197 (5.1)	21.5	24.2	117 (6.0)	20.2	24.3	80 (4.1)	23.5	24.2
Education									
≤High school	748 (19.3)	26.9	26.9	339 (17.4)	25.3	26.0	409 (21.1)	28.3	27.5
College or University	2750 (70.8)	26.7	26.5	1399 (72.0)	25.0	25.7	1351 (69.7)	28.5	27.3
Graduate school	384 (9.9)	25.3	27.0	206 (10.6)	19.8	23.6	178 (9.2)	31.6	29.3
Annual Household income (KRW, million)									
1st Tercile (–30)	1276 (32.9)	29.7	27.4 **	582 (29.9)	29.9	27.5 **	694 (35.8)	29.6	27.4
2nd Tercile (30–50)	1311 (33.8)	25.7	26.1	707 (36.4)	23.3	24.9	604 (31.2)	28.5	27.1
3rd Tercile (51–75)	1295 (33.4)	24.5	26.2	655 (33.7)	21.0	23.7	640 (33.0)	28.0	28.1
Marital status									
Single	1845 (47.5)	28.7	26.9 **	834 (42.9)	27.3	26.7 **	1011 (52.2)	29.9	27.1
Married	1870 (48.2)	24.7	26.3	1051 (54.1)	22.2	24.4	819 (42.3)	27.9	28.2
Separated	46 (1.2)	33.2	30.0	14 (0.7)	41.6	28.5	32 (1.7)	29.5	30.3
Widowed	25 (0.6)	19.4	22.1	6 (0.3)	13.3	19.7	19 (1.0)	21.4	23.0
Divorced	96 (2.5)	21.7	24.0	39 (2.0)	22.5	24.4	57 (2.9)	21.1	23.9
Employment status									
Regular	3480 (89.6)	26.3	26.5	1787 (91.9)	24.0	25.4 *	1693 (87.4)	28.8	27.4
Temporary	310 (8.0)	27.9	27.3	118 (6.1)	28.3	26.2	192 (9.9)	27.7	28.1
Day labourer	92 (2.4)	32.0	30.4	39 (2.0)	34.7	29.8	53 (2.7)	30.1	30.9
Occupation									
White collar	2538 (65.4)	26.3	26.4	1257 (64.7)	23.8	25.3	1281 (66.1)	28.7	27.3
Blue collar	1344 (34.6)	27.2	27.1	687 (35.3)	25.8	26.1	657 (33.9)	28.7	28.0
Weekly working hours									
<40 h/week	552 (14.2)	27.0	27.9	141 (7.3)	24.9	25.4	411 (21.2)	27.7	28.7
=40 h/week	1510 (38.9)	24.9	26.3	651 (33.5)	23.0	25.9	859 (44.3)	26.4	26.5
41–52 h/week	1190 (30.7)	26.8	26.1	711 (36.6)	23.3	24.5	479 (24.7)	32.0	27.6
>52 h/week	630 (16.2)	29.9	27.2	441 (22.7)	28.4	26.6	189 (9.8)	33.2	28.5
Smoking									
No	2878 (74.1)	27.0	26.9	1140 (58.6)	25.1	26.4	1738 (89.7)	28.2	27.2
Yes	1004 (25.9)	25.5	25.9	804 (41.4)	23.6	24.4	200 (10.3)	33.0	30.0
Binge drinking									
No	2204 (56.8)	26.8	26.6	912 (46.9)	25.1	25.8	1292 (66.7)	28.0	27.1
Yes	1678 (43.2)	26.3	26.8	1032 (53.1)	24.0	25.4	646 (33.3)	30.1	28.4
Exercise									
No	2973 (76.6)	27.1	26.6	1375 (70.7)	25.0	25.2	1598 (82.5)	29.0	27.6
Yes	909 (23.4)	24.9	26.9	569 (29.3)	23.3	26.5	340 (17.5)	27.6	27.3

Wilcoxon rank-sum test or Kruskal–Wallis test was performed to test the difference. * *p* < 0.05, ** *p* < 0.001. HRPL: health-related productivity loss. SD: standard deviation.

## Data Availability

The data are not publicly available. The data presented in this study are available on request from the corresponding author.

## Supplementary Materials

The following are available online at https://www.mdpi.com/article/10.3390/ijerph18147589/s1, Figure S1: Histogram of health-related productivity loss among the participants, Figure S2: Percent HRPL according to health conditions, compared to that of participants without any health conditions by gender, Figure S3: Work impairment burden of health conditions compared to that of participants without any health condition (male), Figure S4: Work impairment burden of health conditions compared to that of participants without any health condition (female). Table S1: Demographic characteristics of the study participants by health condition, Table S2: HRPL by health condition, compared to that of participants without any health condition, Table S3: Absenteeism and presenteeism according to symptom, compared to those of participants without any health conditions.
