# Supplementary material for: Health-Related Productivity Loss According to Health Conditions among Workers in South Korea
Source: Int J Environ Res Public Health. 2021 Jul 16;18(14):7589. doi: 10.3390/ijerph18147589 (PMC8307799; doi:10.3390/ijerph18147589)
Supplement: Supplementary file 1 [file ijerph-18-07589-s001.zip › ijerph-1250378-supplementary.pdf]

Table S1. Demographic characteristics of the study participants by health conditions.

	Without any health condition	Fatigue	Neck and/or shoulder pain	Sleep deprivation	Back pain	Headache	Common cold and flu	Eye disorders	Diarrhea or constipation	Insomnia
**Total**	968 (100)	1507 (38.8)	1208 (31.1)	1077 (27.7)	938 (24.2)	838 (21.6)	757 (19.5)	516 (13.3)	442 (11.4)	378 (9.7)
**Mean HRPL (%) (SD)**	16.7 (23.9)	30.5 (26)	32.8 (26.5)	34 (26.3)	33.9 (26.3)	35.3 (27.3)	33.6 (27.9)	32.4 (26.1)	36.2 (27)	40.5 (27.2)
**Gender**										
Male	606 (62.6)	633 (42)	447 (37)	477 (44.3)	382 (40.7)	283 (33.8)	311 (41.1)	155 (30)	167 (37.8)	170 (45)
Female	362 (37.4)	874 (58)	761 (63)	600 (55.7)	556 (59.3)	555 (66.2)	446 (58.9)	361 (70)	275 (62.2)	208 (55)
**Age**										
20~29	238 (24.6)	383 (25.4)	335 (27.7)	313 (29.1)	252 (26.9)	247 (29.5)	193 (25.5)	153 (29.7)	140 (31.7)	101 (26.7)
30~39	244 (25.2)	457 (30.3)	390 (32.3)	365 (33.9)	305 (32.5)	285 (34)	243 (32.1)	151 (29.3)	144 (32.6)	137 (36.2)
40~49	260 (26.9)	442 (29.3)	330 (27.3)	281 (26.1)	255 (27.2)	224 (26.7)	211 (27.9)	123 (23.8)	110 (24.9)	88 (23.3)
50~59	160 (16.5)	168 (11.1)	118 (9.8)	91 (8.4)	88 (9.4)	62 (7.4)	82 (10.8)	71 (13.8)	39 (8.8)	35 (9.3)
60~	66 (6.8)	57 (3.8)	35 (2.9)	27 (2.5)	38 (4.1)	20 (2.4)	28 (3.7)	18 (3.5)	9 (2)	17 (4.5)
**Education**										
≤ High school	196 (20.2)	279 (18.5)	254 (21)	196 (18.2)	206 (22)	155 (18.5)	137 (18.1)	97 (18.8)	78 (17.6)	81 (21.4)
College or University	681 (70.4)	1075 (71.3)	843 (69.8)	774 (71.9)	647 (69)	590 (70.4)	541 (71.5)	365 (70.7)	325 (73.5)	265 (70.1)
Graduate school	91 (9.4)	153 (10.2)	111 (9.2)	107 (9.9)	85 (9.1)	93 (11.1)	79 (10.4)	54 (10.5)	39 (8.8)	32 (8.5)
**Annual Household income (KRW, million)**										
1st Tercile (–30)	311 (32.1)	493 (32.7)	376 (31.1)	372 (34.5)	315 (33.6)	272 (32.5)	261 (34.5)	164 (31.8)	150 (33.9)	143 (37.8)
2nd Tercile (30–50)	328 (33.9)	516 (34.2)	409 (33.9)	345 (32)	314 (33.5)	282 (33.7)	244 (32.2)	180 (34.9)	132 (29.9)	117 (31)
3rd Tercile (51–75)	329 (34)	498 (33)	423 (35)	360 (33.4)	309 (32.9)	284 (33.9)	252 (33.3)	172 (33.3)	160 (36.2)	118 (31.2)
**Marital status**										
Single	447 (46.2)	738 (49)	644 (53.3)	616 (57.2)	487 (51.9)	452 (53.9)	384 (50.7)	297 (57.6)	246 (55.7)	222 (58.7)
Married	485 (50.1)	690 (45.8)	514 (42.6)	415 (38.5)	411 (43.8)	350 (41.8)	343 (45.3)	195 (37.8)	177 (40)	137 (36.2)
Separated	13 (1.3)	23 (1.5)	10 (0.8)	12 (1.1)	7 (0.7)	8 (1)	10 (1.3)	8 (1.6)	5 (1.1)	3 (0.8)
Widowed	2 (0.2)	13 (0.9)	10 (0.8)	9 (0.8)	8 (0.9)	6 (0.7)	5 (0.7)	5 (1)	2 (0.5)	4 (1.1)
Divorced	21 (2.2)	43 (2.9)	30 (2.5)	25 (2.3)	25 (2.7)	22 (2.6)	15 (2)	11 (2.1)	12 (2.7)	12 (3.2)
**Employment status**										
Regular	869 (89.8)	1369 (90.8)	1094 (90.6)	978 (90.8)	839 (89.4)	768 (91.6)	671 (88.6)	476 (92.2)	395 (89.4)	338 (89.4)
Temporary	71 (7.3)	103 (6.8)	91 (7.5)	79 (7.3)	77 (8.2)	58 (6.9)	67 (8.9)	32 (6.2)	36 (8.1)	32 (8.5)
Day labourer	28 (2.9)	35 (2.3)	23 (1.9)	20 (1.9)	22 (2.3)	12 (1.4)	19 (2.5)	8 (1.6)	11 (2.5)	8 (2.1)
**Occupation**										
White collar	611 (63.1)	1017 (67.5)	799 (66.1)	737 (68.4)	572 (61)	585 (69.8)	525 (69.4)	362 (70.2)	288 (65.2)	256 (67.7)
Blue collar	357 (36.9)	490 (32.5)	409 (33.9)	340 (31.6)	366 (39)	253 (30.2)	232 (30.6)	154 (29.8)	154 (34.8)	122 (32.3)
**Weekly working hours**										
< 40 hours / week	137 (14.2)	205 (13.6)	168 (13.9)	131 (12.2)	126 (13.4)	116 (13.8)	120 (15.9)	71 (13.8)	70 (15.8)	51 (13.5)
= 40 hours / week	402 (41.5)	570 (37.8)	460 (38.1)	376 (34.9)	342 (36.5)	323 (38.5)	314 (41.5)	209 (40.5)	163 (36.9)	135 (35.7)
41–52 hours / week	281 (29)	500 (33.2)	383 (31.7)	369 (34.3)	297 (31.7)	268 (32)	215 (28.4)	167 (32.4)	136 (30.8)	130 (34.4)
> 52 hours / week	148 (15.3)	232 (15.4)	197 (16.3)	201 (18.7)	173 (18.4)	131 (15.6)	108 (14.3)	69 (13.4)	73 (16.5)	62 (16.4)

**Smoking**										
No	706 (72.9)	1126 (74.7)	932 (77.2)	784 (72.8)	692 (73.8)	663 (79.1)	562 (74.2)	413 (80)	340 (76.9)	260 (68.8)
Yes	262 (27.1)	381 (25.3)	276 (22.8)	293 (27.2)	246 (26.2)	175 (20.9)	195 (25.8)	103 (20)	102 (23.1)	118 (31.2)
**Binge drinking**										
No	568 (58.7)	834 (55.3)	690 (57.1)	563 (52.3)	531 (56.6)	486 (58)	415 (54.8)	293 (56.8)	230 (52)	190 (50.3)
Yes	400 (41.3)	673 (44.7)	518 (42.9)	514 (47.7)	407 (43.4)	352 (42)	342 (45.2)	223 (43.2)	212 (48)	188 (49.7)
**Exercise**										
No	719 (74.3)	1185 (78.6)	953 (78.9)	824 (76.5)	737 (78.6)	645 (77)	575 (76)	398 (77.1)	333 (75.3)	282 (74.6)
Yes	249 (25.7)	322 (21.4)	255 (21.1)	253 (23.5)	201 (21.4)	193 (23)	182 (24)	118 (22.9)	109 (24.7)	96 (25.4)

	**Anxiety**	**Having a hango-ver**	**Depression**	**Enterocolitis**	**Skin disease or itching**	**Dental problems**	**Allergic disease**	**Respiratory symptoms**	**Urinary symp-toms**	**Anemia**
**Total**	358 (9.2)	303 (7.8)	296 (7.6)	325 (8.4)	279 (7.2)	213 (5.5)	203 (5.2)	175 (4.5)	175 (4.5)	147 (3.8)
**Mean HRPL (%) (SD)**	41.2 (26.7)	32 (27.2)	41.3 (25.8)	40.5 (28.2)	33.3 (27.7)	33.2 (25.6)	34.1 (27.5)	39.2 (30.1)	35.2 (27.2)	40.7 (27.4)
**Gender**										
Male	161 (45)	211 (64.9)	110 (36.3)	131 (44.3)	114 (40.9)	99 (46.5)	58 (28.6)	85 (48.6)	72 (41.1)	33 (22.4)
Female	197 (55)	114 (35.1)	193 (63.7)	165 (55.7)	165 (59.1)	114 (53.5)	145 (71.4)	90 (51.4)	103 (58.9)	114 (77.6)
**Age**										
20~29	104 (29.1)	86 (26.5)	105 (34.7)	90 (30.4)	81 (29)	41 (19.2)	47 (23.2)	49 (28)	40 (22.9)	50 (34)
30~39	126 (35.2)	106 (32.6)	103 (34)	109 (36.8)	95 (34.1)	67 (31.5)	61 (30)	68 (38.9)	49 (28)	39 (26.5)
40~49	94 (26.3)	96 (29.5)	72 (23.8)	75 (25.3)	71 (25.4)	62 (29.1)	64 (31.5)	39 (22.3)	54 (30.9)	38 (25.9)
50~59	25 (7)	27 (8.3)	17 (5.6)	18 (6.1)	22 (7.9)	27 (12.7)	25 (12.3)	12 (6.9)	13 (7.4)	17 (11.6)
60~	9 (2.5)	10 (3.1)	6 (2)	4 (1.4)	10 (3.6)	16 (7.5)	6 (3)	7 (4)	19 (10.9)	3 (2)
**Education**										
≤ High school	70 (19.6)	47 (14.5)	65 (21.5)	44 (14.9)	49 (17.6)	39 (18.3)	36 (17.7)	34 (19.4)	28 (16)	35 (23.8)
College or University	253 (70.7)	253 (77.8)	212 (70)	222 (75)	191 (68.5)	148 (69.5)	145 (71.4)	126 (72)	128 (73.1)	103 (70.1)
Graduate school	35 (9.8)	25 (7.7)	26 (8.6)	30 (10.1)	39 (14)	26 (12.2)	22 (10.8)	15 (8.6)	19 (10.9)	9 (6.1)
**Annual Household income (KRW, million)**										
1st Tercile (–30)	144 (40.2)	102 (31.4)	121 (39.9)	104 (35.1)	97 (34.8)	73 (34.3)	71 (35)	60 (34.3)	72 (41.1)	56 (38.1)
2nd Tercile (30–50)	114 (31.8)	112 (34.5)	98 (32.3)	101 (34.1)	92 (33)	69 (32.4)	59 (29.1)	57 (32.6)	52 (29.7)	44 (29.9)
3rd Tercile (51–75)	100 (27.9)	111 (34.2)	84 (27.7)	91 (30.7)	90 (32.3)	71 (33.3)	73 (36)	58 (33.1)	51 (29.1)	47 (32)
**Marital status**										
Single	222 (62)	164 (50.5)	194 (64)	170 (57.4)	155 (55.6)	99 (46.5)	105 (51.7)	97 (55.4)	79 (45.1)	83 (56.5)
Married	121 (33.8)	152 (46.8)	96 (31.7)	114 (38.5)	113 (40.5)	105 (49.3)	88 (43.4)	67 (38.3)	88 (50.3)	55 (37.4)
Separated	2 (0.6)	1 (0.3)	1 (0.3)	1 (0.3)	4 (1.4)	2 (0.9)	1 (0.5)	2 (1.1)	3 (1.7)	3 (2)
Widowed	5 (1.4)	1 (0.3)	2 (0.7)	1 (0.3)	2 (0.7)	2 (0.9)	0 (0)	3 (1.7)	0 (0)	1 (0.7)
Divorced	8 (2.2)	7 (2.2)	10 (3.3)	10 (3.4)	5 (1.8)	5 (2.3)	9 (4.4)	6 (3.4)	5 (2.9)	5 (3.4)
**Employment status**										
Regular	321 (89.7)	303 (93.2)	266 (87.8)	267 (90.2)	254 (91)	195 (91.5)	182 (89.7)	148 (84.6)	155 (88.6)	132 (89.8)
Temporary	23 (6.4)	19 (5.8)	27 (8.9)	22 (7.4)	23 (8.2)	17 (8)	19 (9.4)	23 (13.1)	15 (8.6)	12 (8.2)
Day labourer	14 (3.9)	3 (0.9)	10 (3.3)	7 (2.4)	2 (0.7)	1 (0.5)	2 (1)	4 (2.3)	5 (2.9)	3 (2)
**Occupation**										
White collar	236 (65.9)	229 (70.5)	206 (68)	212 (71.6)	172 (61.6)	149 (70)	140 (69)	94 (53.7)	108 (61.7)	93 (63.3)

Blue collar	122 (34.1)	96 (29.5)	97 (32)	84 (28.4)	107 (38.4)	64 (30)	63 (31)	81 (46.3)	67 (38.3)	54 (36.7)
**Weekly working hours**										
< 40 hours / week	43 (12)	27 (8.3)	48 (15.8)	37 (12.5)	37 (13.3)	28 (13.1)	31 (15.3)	26 (14.9)	29 (16.6)	23 (15.6)
= 40 hours / week	125 (34.9)	114 (35.1)	105 (34.7)	116 (39.2)	106 (38)	77 (36.2)	72 (35.5)	55 (31.4)	66 (37.7)	53 (36.1)
41–52 hours / week	118 (33)	113 (34.8)	101 (33.3)	87 (29.4)	84 (30.1)	70 (32.9)	67 (33)	55 (31.4)	58 (33.1)	50 (34)
> 52 hours / week	72 (20.1)	71 (21.8)	49 (16.2)	56 (18.9)	52 (18.6)	38 (17.8)	33 (16.3)	39 (22.3)	22 (12.6)	21 (14.3)
**Smoking**										
No	270 (75.4)	177 (54.5)	232 (76.6)	216 (73)	203 (72.8)	152 (71.4)	163 (80.3)	122 (69.7)	148 (84.6)	112 (76.2)
Yes	88 (24.6)	148 (45.5)	71 (23.4)	80 (27)	76 (27.2)	61 (28.6)	40 (19.7)	53 (30.3)	27 (15.4)	35 (23.8)
**Binge drinking**										
No	190 (53.1)	52 (16)	162 (53.5)	146 (49.3)	148 (53)	113 (53.1)	108 (53.2)	93 (53.1)	107 (61.1)	90 (61.2)
Yes	168 (46.9)	273 (84)	141 (46.5)	150 (50.7)	131 (47)	100 (46.9)	95 (46.8)	82 (46.9)	68 (38.9)	57 (38.8)
**Exercise**										
No	263 (73.5)	238 (73.2)	240 (79.2)	231 (78)	224 (80.3)	161 (75.6)	159 (78.3)	141 (80.6)	141 (80.6)	117 (79.6)
Yes	95 (26.5)	87 (26.8)	63 (20.8)	65 (22)	55 (19.7)	52 (24.4)	44 (21.7)	34 (19.4)	34 (19.4)	30 (20.4)

	Esophagitis	Hypertension	Gastric ulcer or duodenal ulcer	Upper limb or lower limb pain	Dyslipidemia	Post-menopausal or post-andropausal symptoms	Hearing impairment	Asthma	Diabetes mellitus	Etc.
**Total**	144 (3.7)	117 (3)	119 (3.1)	106 (2.7)	91 (2.3)	83 (2.1)	80 (2.1)	47 (1.2)	51 (1.3)	47 (1.2)
**Mean HRPL (%) (SD)**	38.5 (26.7)	33.5 (26)	41.7 (31)	36.9 (27.4)	37.2 (26.3)	32 (28.6)	40 (26.6)	32.2 (25.7)	34.9 (28.6)	28.8 (25)
**Gender**										
Male	62 (43.1)	96 (80.7)	50 (42.7)	35 (33)	65 (71.4)	16 (20)	53 (63.9)	32 (62.7)	35 (74.5)	15 (31.9)
Female	82 (56.9)	23 (19.3)	67 (57.3)	71 (67)	26 (28.6)	64 (80)	30 (36.1)	19 (37.3)	12 (25.5)	32 (68.1)
**Age**										
20~29	35 (24.3)	10 (8.4)	12 (10.3)	23 (21.7)	1 (1.1)	0 (0)	29 (34.9)	9 (17.6)	6 (12.8)	11 (23.4)
30~39	43 (29.9)	19 (16)	42 (35.9)	25 (23.6)	11 (12.1)	2 (2.5)	23 (27.7)	15 (29.4)	4 (8.5)	21 (44.7)
40~49	48 (33.3)	37 (31.1)	46 (39.3)	35 (33)	34 (37.4)	25 (31.3)	16 (19.3)	17 (33.3)	12 (25.5)	7 (14.9)
50~59	16 (11.1)	30 (25.2)	12 (10.3)	19 (17.9)	29 (31.9)	43 (53.8)	11 (13.3)	5 (9.8)	15 (31.9)	7 (14.9)
60~	2 (1.4)	23 (19.3)	5 (4.3)	4 (3.8)	16 (17.6)	10 (12.5)	4 (4.8)	5 (9.8)	10 (21.3)	1 (2.1)
**Education**										
≤ High school	27 (18.8)	27 (22.7)	15 (12.8)	25 (23.6)	11 (12.1)	22 (27.5)	22 (26.5)	11 (21.6)	6 (12.8)	11 (23.4)
College or University	96 (66.7)	82 (68.9)	83 (70.9)	69 (65.1)	63 (69.2)	45 (56.3)	55 (66.3)	36 (70.6)	32 (68.1)	36 (76.6)
Graduate school	21 (14.6)	10 (8.4)	19 (16.2)	12 (11.3)	17 (18.7)	13 (16.3)	6 (7.2)	4 (7.8)	9 (19.1)	0 (0)
**Annual Household income (KRW, million)**										
1st Tercile (–30)	36 (25)	35 (29.4)	35 (29.9)	34 (32.1)	18 (19.8)	28 (35)	29 (34.9)	21 (41.2)	12 (25.5)	12 (25.5)
2nd Tercile (30–50)	54 (37.5)	53 (44.5)	37 (31.6)	43 (40.6)	33 (36.3)	28 (35)	33 (39.8)	13 (25.5)	19 (40.4)	18 (38.3)
3rd Tercile (51–75)	54 (37.5)	31 (26.1)	45 (38.5)	29 (27.4)	40 (44)	24 (30)	21 (25.3)	17 (33.3)	16 (34)	17 (36.2)
**Marital status**										
Single	65 (45.1)	29 (24.4)	48 (41)	45 (42.5)	16 (17.6)	11 (13.8)	51 (61.4)	20 (39.2)	14 (29.8)	28 (59.6)
Married	75 (52.1)	80 (67.2)	66 (56.4)	58 (54.7)	72 (79.1)	57 (71.3)	30 (36.1)	28 (54.9)	31 (66)	18 (38.3)
Separated	1 (0.7)	0 (0)	1 (0.9)	0 (0)	1 (1.1)	0 (0)	0 (0)	0 (0)	0 (0)	1 (2.1)
Widowed	0 (0)	2 (1.7)	0 (0)	2 (1.9)	0 (0)	5 (6.3)	0 (0)	1 (2)	0 (0)	0 (0)

Divorced	3 (2.1)	8 (6.7)	2 (1.7)	1 (0.9)	2 (2.2)	7 (8.8)	2 (2.4)	2 (3.9)	2 (4.3)	0 (0)
**Employment status**										
Regular	129 (89.6)	109 (91.6)	104 (88.9)	92 (86.8)	81 (89)	69 (86.3)	68 (81.9)	45 (88.2)	44 (93.6)	43 (91.5)
Temporary	13 (9)	7 (5.9)	10 (8.5)	12 (11.3)	9 (9.9)	11 (13.8)	13 (15.7)	6 (11.8)	3 (6.4)	2 (4.3)
Day labourer	2 (1.4)	3 (2.5)	3 (2.6)	2 (1.9)	1 (1.1)	0 (0)	2 (2.4)	0 (0)	0 (0)	2 (4.3)
**Occupation**										
White collar	89 (61.8)	73 (61.3)	76 (65)	44 (41.5)	59 (64.8)	46 (57.5)	39 (47)	36 (70.6)	32 (68.1)	34 (72.3)
Blue collar	55 (38.2)	46 (38.7)	41 (35)	62 (58.5)	32 (35.2)	34 (42.5)	44 (53)	15 (29.4)	15 (31.9)	13 (27.7)
**Weekly working hours**										
< 40 hours / week	16 (11.1)	16 (13.4)	14 (12)	15 (14.2)	11 (12.1)	15 (18.8)	14 (16.9)	6 (11.8)	9 (19.1)	9 (19.1)
= 40 hours / week	54 (37.5)	35 (29.4)	35 (29.9)	42 (39.6)	26 (28.6)	37 (46.3)	33 (39.8)	19 (37.3)	22 (46.8)	18 (38.3)
41–52 hours / week	49 (34)	44 (37)	48 (41)	32 (30.2)	31 (34.1)	14 (17.5)	18 (21.7)	14 (27.5)	9 (19.1)	14 (29.8)
> 52 hours / week	25 (17.4)	24 (20.2)	20 (17.1)	17 (16)	23 (25.3)	14 (17.5)	18 (21.7)	12 (23.5)	7 (14.9)	6 (12.8)
**Smoking**										
No	101 (70.1)	72 (60.5)	95 (81.2)	82 (77.4)	54 (59.3)	70 (87.5)	59 (71.1)	34 (66.7)	18 (38.3)	40 (85.1)
Yes	43 (29.9)	47 (39.5)	22 (18.8)	24 (22.6)	37 (40.7)	10 (12.5)	24 (28.9)	17 (33.3)	29 (61.7)	7 (14.9)
**Binge drinking**										
No	79 (54.9)	55 (46.2)	63 (53.8)	66 (62.3)	48 (52.7)	53 (66.3)	48 (57.8)	24 (47.1)	21 (44.7)	30 (63.8)
Yes	65 (45.1)	64 (53.8)	54 (46.2)	40 (37.7)	43 (47.3)	27 (33.8)	35 (42.2)	27 (52.9)	26 (55.3)	17 (36.2)
**Exercise**										
No	105 (72.9)	93 (78.2)	85 (72.7)	87 (82.1)	71 (78)	67 (83.8)	64 (77.1)	35 (68.6)	36 (76.6)	32 (68.1)
Yes	39 (27.1)	26 (21.8)	32 (27.4)	19 (17.9)	20 (22)	13 (16.3)	19 (22.9)	16 (31.4)	11 (23.4)	15 (31.9)

	Liver disease	Cardiac disease	Osteoporosis	Cancer	Infertility treatment
**Total**	29 (0.7)	27 (0.7)	32 (0.8)	21 (0.5)	11 (0.3)
**Mean HRPL (%) (SD)**	30.6 (28.4)	38.2 (23.2)	41 (30.5)	40.9 (31.4)	49.2 (23.2)
**Gender**					
Male	26 (81.3)	23 (79.3)	3 (11.1)	14 (66.7)	3 (27.3)
Female	6 (18.8)	6 (20.7)	24 (88.9)	7 (33.3)	8 (72.7)
**Age**					
20~29	7 (21.9)	6 (20.7)	6 (22.2)	3 (14.3)	2 (18.2)
30~39	7 (21.9)	2 (6.9)	4 (14.8)	3 (14.3)	5 (45.5)
40~49	11 (34.4)	9 (31)	4 (14.8)	7 (33.3)	3 (27.3)
50~59	5 (15.6)	9 (31)	8 (29.6)	8 (38.1)	1 (9.1)
60~	2 (6.3)	3 (10.3)	5 (18.5)	0 (0)	0 (0)
**Education**					
≤ High school	5 (15.6)	8 (27.6)	9 (33.3)	2 (9.5)	0 (0)
College or University	25 (78.1)	18 (62.1)	14 (51.9)	17 (81)	7 (63.6)
Graduate school	2 (6.3)	3 (10.3)	4 (14.8)	2 (9.5)	4 (36.4)
**Annual Household income (KRW, million)**					
1st Tercile (–30)	10 (31.3)	6 (20.7)	10 (37)	6 (28.6)	4 (36.4)
2nd Tercile (30–50)	7 (21.9)	8 (27.6)	9 (33.3)	4 (19)	3 (27.3)

3rd Tercile (51–75)	15 (46.9)	15 (51.7)	8 (29.6)	11 (52.4)	4 (36.4)
**Marital status**					
Single	12 (37.5)	8 (27.6)	9 (33.3)	4 (19)	2 (18.2)
Married	19 (59.4)	19 (65.5)	14 (51.9)	16 (76.2)	8 (72.7)
Separated	0 (0)	0 (0)	0 (0)	1 (4.8)	1 (9.1)
Widowed	0 (0)	0 (0)	2 (7.4)	0 (0)	0 (0)
Divorced	1 (3.1)	2 (6.9)	2 (7.4)	0 (0)	0 (0)
**Employment status**					
Regular	28 (87.5)	27 (93.1)	24 (88.9)	21 (100)	8 (72.7)
Temporary	4 (12.5)	1 (3.4)	2 (7.4)	0 (0)	3 (27.3)
Day labourer	0 (0)	1 (3.4)	1 (3.7)	0 (0)	0 (0)
**Occupation**					
White collar	24 (75)	16 (55.2)	13 (48.1)	15 (71.4)	9 (81.8)
Blue collar	8 (25)	13 (44.8)	14 (51.9)	6 (28.6)	2 (18.2)
**Weekly working hours**					
< 40 hours / week	3 (9.4)	2 (6.9)	8 (29.6)	4 (19)	2 (18.2)
= 40 hours / week	10 (31.3)	11 (37.9)	8 (29.6)	9 (42.9)	4 (36.4)
41–52 hours / week	12 (37.5)	8 (27.6)	6 (22.2)	5 (23.8)	3 (27.3)
> 52 hours / week	7 (21.9)	8 (27.6)	5 (18.5)	3 (14.3)	2 (18.2)
**Smoking**					
No	14 (43.8)	21 (72.4)	22 (81.5)	18 (85.7)	9 (81.8)
Yes	18 (56.3)	8 (27.6)	5 (18.5)	3 (14.3)	2 (18.2)
**Binge drinking**					
No	14 (43.8)	19 (65.5)	17 (63)	15 (71.4)	9 (81.8)
Yes	18 (56.3)	10 (34.5)	10 (37)	6 (28.6)	2 (18.2)
**Exercise**					
No	25 (78.1)	19 (65.5)	24 (88.9)	14 (66.7)	10 (90.9)
Yes	7 (21.9)	10 (34.5)	3 (11.1)	7 (33.3)	1 (9.1)

Table S2. HRPL by the health conditions, comparing to the participants without any health conditions.

Health Condition	Total				Male				Female			
	n (%)	Rank	The ratio of HRPL (vs. without any health conditions) *	*p*-value	n (%)	Rank	The ratio of HRPL (vs. without any health conditions) *	*p*-value	n (%)	Rank	The ratio of HRPL (vs. without any health conditions) *	*p*-value
Fatigue	1507 (38.8)	1	1.79	**<.0001**	633 (32.6)	1	1.72	**<.0001**	874 (45.1)	1	1.96	**<.0001**
Neck and/or shoulder pain	1208 (31.1)	2	1.92	**<.0001**	447 (23)	3	1.86	**<.0001**	761 (39.3)	2	2.03	**<.0001**
Sleep deprivation	1077 (27.7)	3	1.96	**<.0001**	477 (24.5)	2	1.83	**<.0001**	600 (31)	3	2.18	**<.0001**
Back pain	938 (24.2)	4	2.00	**<.0001**	382 (19.7)	4	1.87	**<.0001**	556 (28.7)	4	2.20	**<.0001**
Headache	838 (21.6)	5	2.06	**<.0001**	283 (14.6)	6	1.98	**<.0001**	555 (28.6)	5	2.18	**<.0001**
Common cold and flu	757 (19.5)	6	1.98	**<.0001**	311 (16)	5	1.95	**<.0001**	446 (23)	6	2.08	**<.0001**
Eye disorders	516 (13.3)	7	1.95	**<.0001**	155 (8)	11	2.00	**0.0002**	361 (18.6)	7	1.94	**<.0001**
Diarrhea or constipation	442 (11.4)	8	2.14	**<.0001**	167 (8.6)	9	2.01	**0.0001**	275 (14.2)	8	2.24	**<.0001**
Insomnia	378 (9.7)	9	2.39	**<.0001**	170 (8.7)	8	2.33	**<.0001**	208 (10.7)	9	2.53	**<.0001**
Anxiety	358 (9.2)	10	2.46	**<.0001**	161 (8.3)	10	2.40	**<.0001**	197 (10.2)	10	2.62	**<.0001**
Having a hangover	303 (7.8)	12	2.42	**<.0001**	211 (10.9)	7	2.02	**<.0001**	114 (5.9)	15	1.88	0.0075
Depression	296 (7.6)	13	2.38	**<.0001**	110 (5.7)	14	2.40	**<.0001**	193 (10)	11	2.48	**<.0001**
Enterocolitis	325 (8.4)	11	1.93	**<.0001**	131 (6.7)	12	2.43	**<.0001**	165 (8.5)	12	2.44	**<.0001**
Skin disease or itching	279 (7.2)	14	1.94	**<.0001**	114 (5.9)	13	1.78	0.0093	165 (8.5)	12	2.13	**<.0001**
Dental problems	213 (5.5)	15	2.01	**<.0001**	99 (5.1)	15	1.91	0.0063	114 (5.9)	15	2.24	**0.0004**
Allergic disease	203 (5.2)	16	2.04	**<.0001**	58 (3)	21	1.99	0.0296	145 (7.5)	14	2.13	**0.0002**
Respiratory symptoms	175 (4.5)	17	2.25	**<.0001**	85 (4.4)	17	2.13	0.0036	90 (4.6)	19	2.52	**0.0003**
Urinary symptoms	175 (4.5)	17	2.14	**<.0001**	72 (3.7)	18	2.20	0.0046	103 (5.3)	18	1.93	0.0073
Anemia	147 (3.8)	19	2.47	**<.0001**	33 (1.7)	26	2.50	0.0260	114 (5.9)	15	2.46	**<.0001**
Esophagitis	144 (3.7)	20	2.36	**<.0001**	62 (3.2)	20	2.19	0.0102	82 (4.2)	20	2.65	**0.0002**
Hypertension	117 (3)	22	2.56	**0.0002**	96 (4.9)	16	2.24	**0.0011**	23 (1.2)	28	2.72	0.0617
Gastric ulcer or duodenal ulcer	119 (3.1)	21	2.28	**<.0001**	50 (2.6)	23	2.66	0.0039	67 (3.5)	22	2.54	0.0017
Upper limb or lower limb pain	106 (2.7)	23	2.34	**0.0003**	35 (1.8)	24	2.57	0.0192	71 (3.7)	21	2.31	0.0039
Dyslipidemia	91 (2.3)	24	2.58	**0.0002**	65 (3.3)	19	2.72	**0.0007**	26 (1.3)	26	2.58	0.0578
Post-menopausal or post-andropausal symptoms	83 (2.1)	25	2.47	0.0020	16 (0.8)	30	2.74	0.0906	64 (3.3)	23	2.58	0.0076
Hearing impairment	80 (2.1)	26	2.41	**0.0007**	53 (2.7)	22	2.10	0.0216	30 (1.6)	25	2.74	0.0217
Asthma	47 (1.2)	28	2.32	0.0378	32 (1.7)	27	1.76	0.1833	19 (1)	29	2.47	0.1038

Health Condition	Total				Male				Female			
	n (%)	Rank	The ratio of HRPL (vs. without any health conditions) *	*p*-value	n (%)	Rank	The ratio of HRPL (vs. without any health conditions) *	*p*-value	n (%)	Rank	The ratio of HRPL (vs. without any health conditions) *	*p*-value
Diabetes mellitus	51 (1.3)	27	2.00	0.0166	35 (1.8)	24	2.15	0.0607	12 (0.6)	30	2.90	0.1313
Etc.	47 (1.2)	28	1.60	0.1901	15 (0.8)	31	1.25	0.7218	32 (1.7)	24	1.87	0.1557
Liver disease	29 (0.7)	31	2.55	0.1050	26 (1.3)	28	2.02	0.1313	6 (0.3)	33	2.07	0.4786
Cardiac disease	27 (0.7)	32	2.67	0.0336	23 (1.2)	29	2.72	0.0410	6 (0.3)	33	2.75	0.3069
Osteoporosis	32 (0.8)	30	1.99	0.0435	3 (0.2)	33	2.05	0.6057	24 (1.2)	27	2.74	0.0516
Cancer	21 (0.5)	33	2.71	0.0562	14 (0.7)	32	2.28	0.1924	7 (0.4)	32	4.40	0.1122
Infertility treatment	11 (0.3)	34	2.86	0.1471	3 (0.2)	33	2.32	0.5407	8 (0.4)	31	2.82	0.2292

* The ratio of HRPL of participants with a specific health condition to HRPL of participants without any health conditions, calculated by negative binomial regression models, adjusted for age, gender, education level, household income, marital status, employment status, and weekly working hours. HRPL: health-related productivity loss, $p < 0.0014$ was in bold.

Table S3. Absenteeism and presenteeism according to symptom, comparing to the participants without any health conditions.

Health Conditions	n (%)	Rank	Absenteeism			Presenteeism		
			**The ratio of absenteeism (vs. without any health conditions) ***	***p*-value**	**Rank**	**The ratio of presenteeism (vs. without any health conditions) ***	***p*-value**	**Rank**
Fatigue	1507 (38.8)	1	2.19	0.0018	33	1.80	**<.0001**	33
Neck and/or shoulder pain	1208 (31.1)	2	2.26	0.0022	31	1.93	**<.0001**	31
Sleep deprivation	1077 (27.7)	3	2.28	0.0030	30	1.97	**<.0001**	30
Back pain	938 (24.2)	4	2.49	0.0018	28	2.01	**<.0001**	28
Headache	838 (21.6)	5	**2.77**	**0.0002**	21	2.06	**<.0001**	21
Common cold and flu	757 (19.5)	6	**3.16**	**<0.0001**	18	1.98	**<.0001**	18
Eye disorders	516 (13.3)	7	2.64	0.0045	25	1.95	**<.0001**	25
Diarrhea or constipation	442 (11.4)	8	2.24	0.0232	32	2.13	**<.0001**	32
Insomnia	378 (9.7)	9	**3.69**	**0.0005**	14	2.39	**<.0001**	14
Anxiety	358 (9.2)	10	**4.03**	**0.0004**	13	2.47	**<.0001**	13
Having a hangover	303 (7.8)	12	2.66	0.0353	24	1.90	**<.0001**	24
Depression	296 (7.6)	13	2.85	0.0184	20	2.43	**<.0001**	20
Enterocolitis	325 (8.4)	11	3.10	0.0063	19	2.41	**<.0001**	19
Skin disease or itching	279 (7.2)	14	2.52	0.0506	26	1.96	**<.0001**	26
Dental problems	213 (5.5)	15	2.72	0.0611	22	2.01	**<.0001**	22
Allergic disease	203 (5.2)	16	2.71	0.0773	23	2.05	**<.0001**	23
Respiratory symptoms	175 (4.5)	17	5.09	0.0040	7	2.22	**<.0001**	7
Urinary symptoms	175 (4.5)	17	2.52	0.1537	27	2.16	**<.0001**	27
Anemia	147 (3.8)	19	4.69	0.0202	8	2.44	**<.0001**	8
Esophagitis	144 (3.7)	20	3.18	0.0952	17	2.39	**<.0001**	17
Hypertension	117 (3)	22	5.13	0.0136	6	2.24	**0.0004**	6
Gastric ulcer or duodenal ulcer	119 (3.1)	21	4.51	0.0310	10	2.58	**<.0001**	10
Upper limb or lower limb pain	106 (2.7)	23	3.35	0.1157	16	2.36	**0.0003**	16
Dyslipidemia	91 (2.3)	24	5.79	0.0144	5	2.53	**0.0004**	5
Post-menopausal or post-andropausal symptoms	83 (2.1)	25	3.62	0.1590	15	2.45	0.0026	15
Hearing impairment	80 (2.1)	26	4.24	0.0966	12	2.40	**0.0009**	12
Asthma	47 (1.2)	28	4.30	0.1999	11	1.96	0.0486	11
Diabetes mellitus	51 (1.3)	27	6.56	0.0965	4	2.08	0.0419	4
Etc.	47 (1.2)	28	0.04	0.0457	34	1.65	0.1696	34
Liver disease	29 (0.7)	31	13.46	0.0645	3	1.72	0.2120	3
Cardiac disease	27 (0.7)	32	2.37	0.5511	29	2.57	0.0359	29
Osteoporosis	32 (0.8)	30	4.55	0.3567	9	2.64	0.0505	9
Cancer	21 (0.5)	33	28.84	0.0903	1	2.70	0.0625	1
Infertility treatment	11 (0.3)	34	14.97	0.4629	2	2.93	0.1456	2

* The ratio of absenteeism and presenteeism of participants with a specific health condition to HRPL of participants without any health conditions, calculated by negative binomial regression models, adjusted for age, gender, education level, household income, marital status, employment status, and weekly working hours. HRPL: health-related productivity loss, $p < 0.0014$ was in bold.

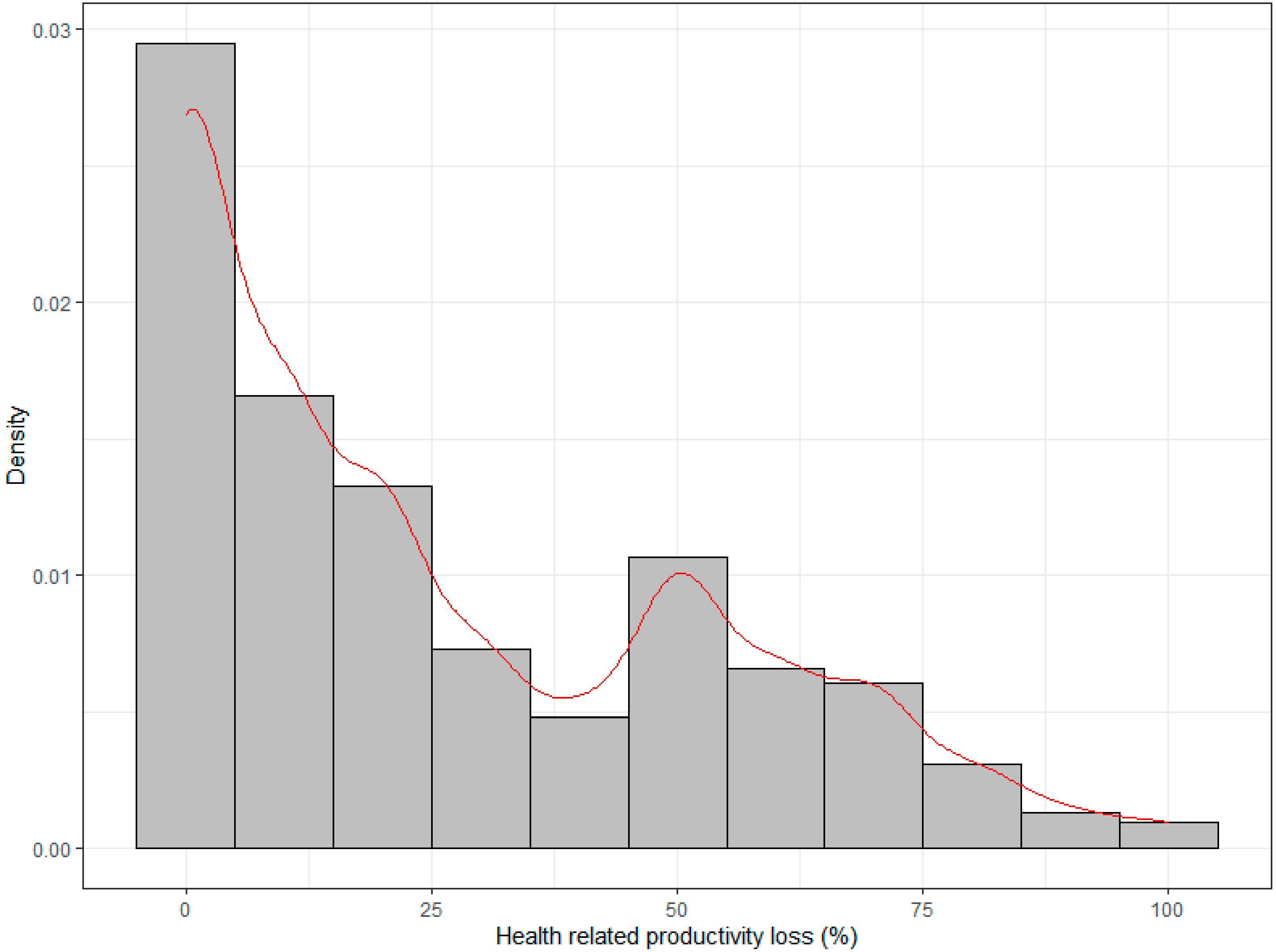

Figure S1. Histogram of health-related productivity loss among the participants.

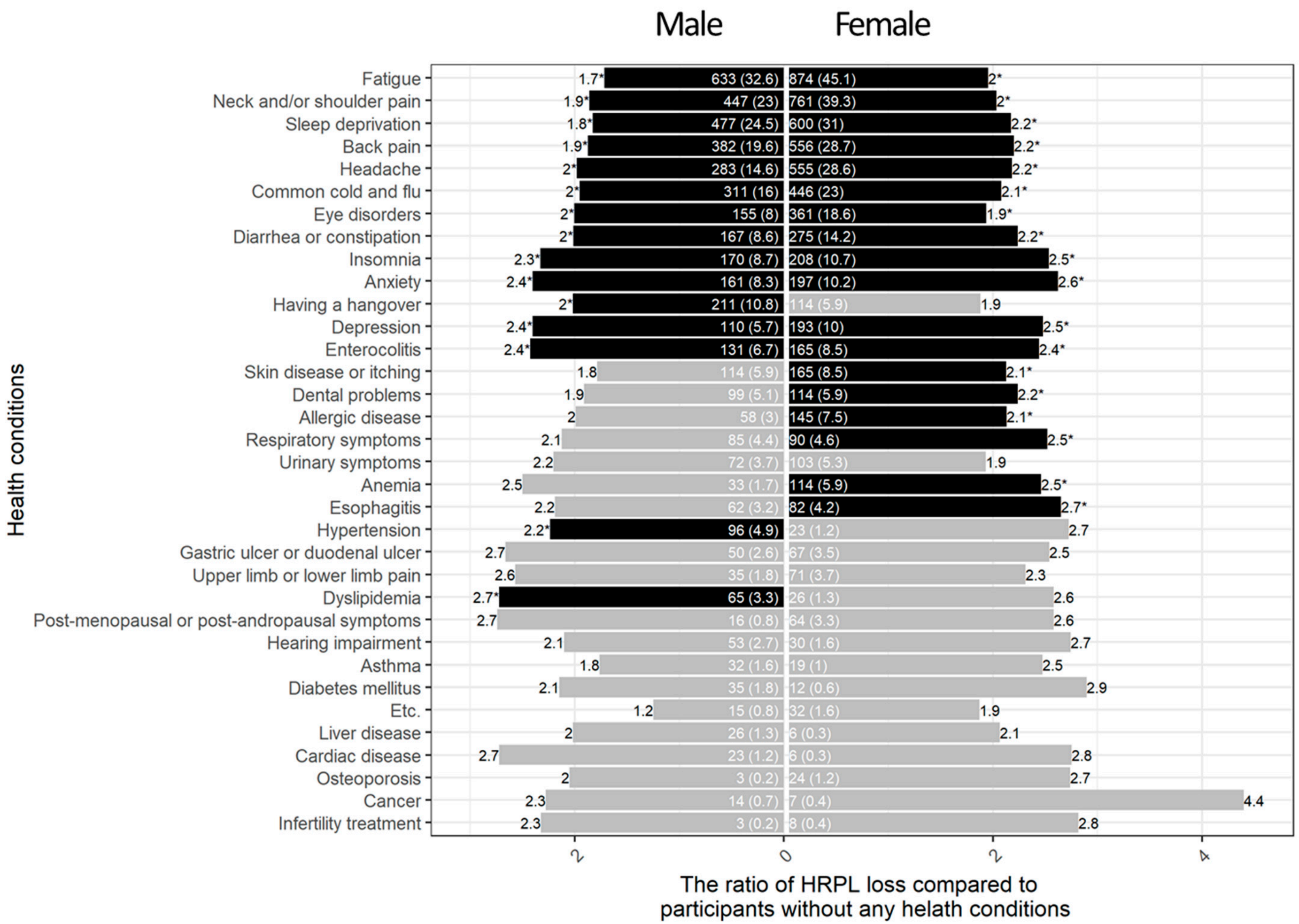

Figure S2. Percent HRPL according to health conditions, comparing to the participants without any health conditions by gender.

Percent health-related productivity loss among workers with specific health conditions, comparing to the participants without any health conditions. The number of workers and percent who complaint specific health conditions were presented in the bottom of the bar plot. The ratio of HRPL loss of participants with specific health conditions to participants without any health conditions was calculated with the adjustment for age, gender, education level, household income, marital status, employment status, and weekly working hours. * $p < 0.0014$ was in black.

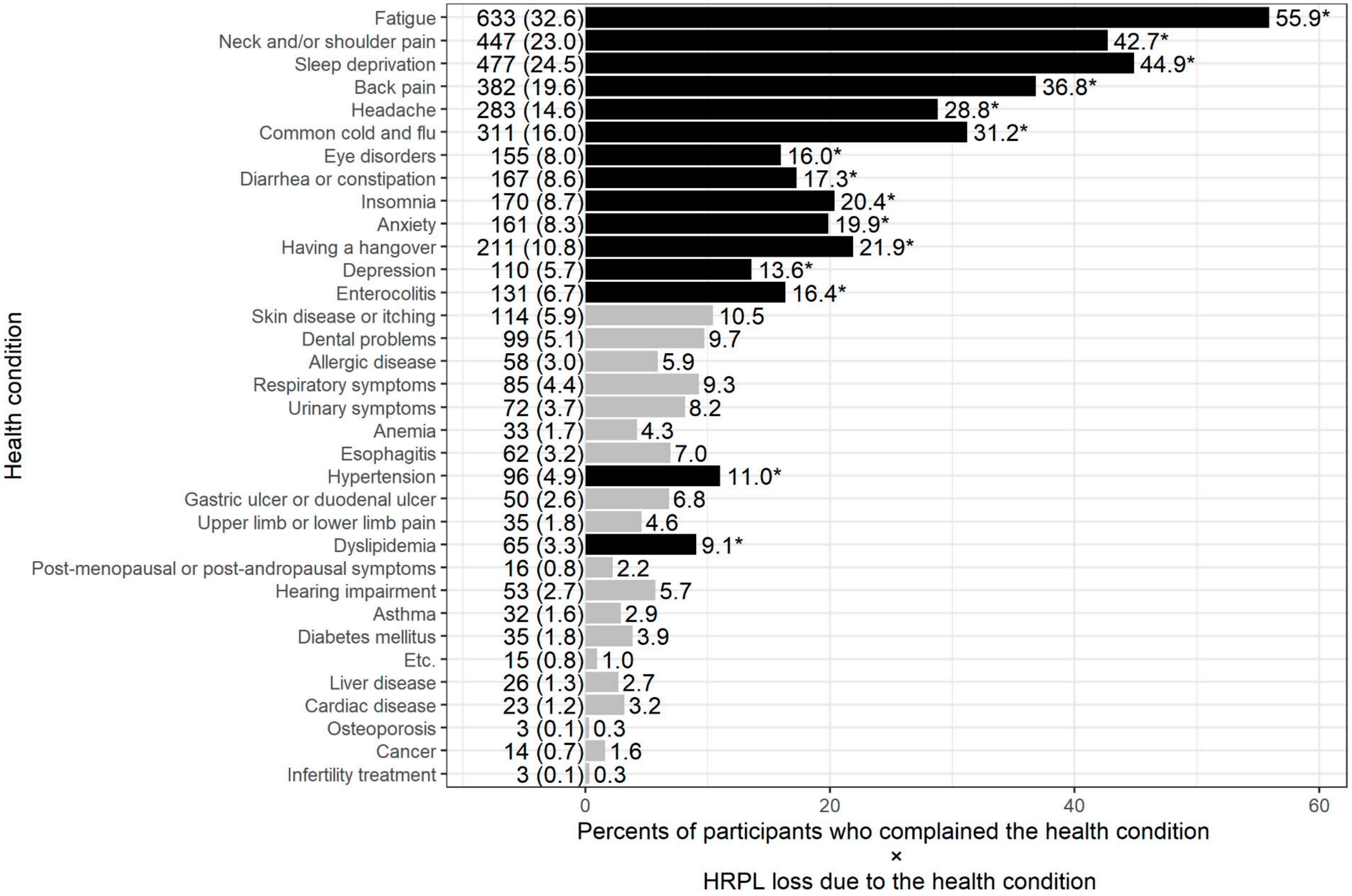

Figure S3. Work impairment burden of health conditions comparing to the participants without any health conditions (male).

Percent health-related productivity loss among workers with specific health conditions, comparing to the participants without any health conditions. The number of workers and percent who complaint specific health conditions were presented in the bottom of the bar plot The ratio of HRPL loss of participants with specific health conditions to participants without any health conditions was calculated with the adjustment for age, gender, education level, household income, marital status, employment status, and weekly working hours. * $p < 0.0014$ was in black.

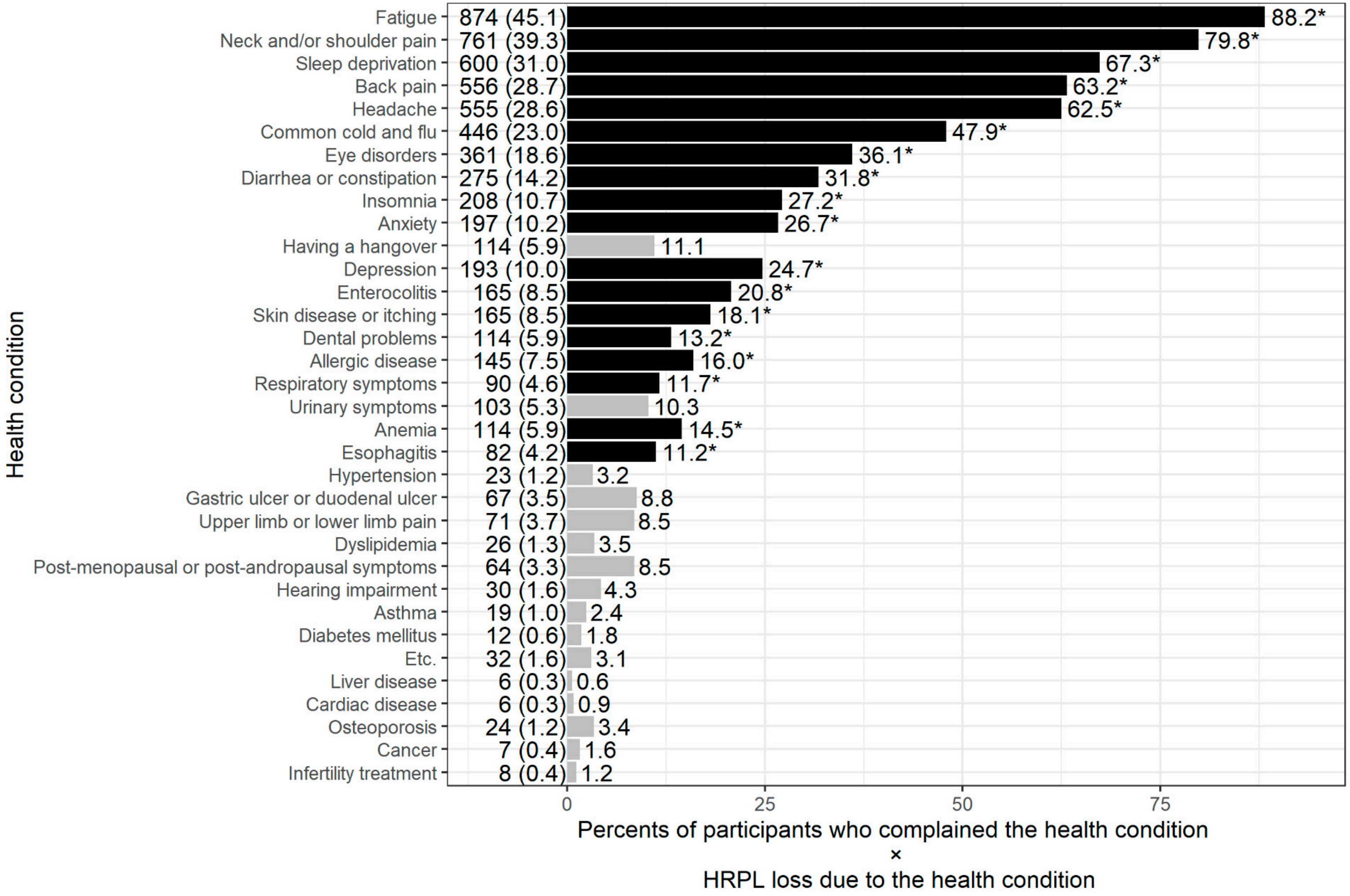

Figure S4. Work impairment burden of health conditions comparing to the participants without any health conditions (female).

Percent health-related productivity loss among workers with specific health conditions, comparing to the participants without any health conditions. The number of workers and percent who complaint specific health conditions were presented in the bottom of the bar plot. The ratio of HRPL loss of participants with specific health conditions to participants without any health conditions was calculated with the adjustment for age, gender, education level, household income, marital status, employment status, and weekly working hours. * $p < 0.0014$ was in black.
